# Overview of Cardiomyopathies in Childhood

**DOI:** 10.3389/fped.2021.708732

**Published:** 2021-07-23

**Authors:** Anika Rath, Robert Weintraub

**Affiliations:** ^1^Department of Cardiology, Royal Children's Hospital, Melbourne, VIC, Australia; ^2^Heart Research, Murdoch Children's Research Institute, Melbourne, VIC, Australia; ^3^Department of Paediatrics, Melbourne University, Melbourne, VIC, Australia

**Keywords:** cardiomyopathy, paediatric, epidemiology, long-term outcomes, risk factors, sudden cardiac death, heart transplantation

## Abstract

Paediatric cardiomyopathies are a heterogenous group of rare disorders, characterised by mechanical and electrical abnormalities of the heart muscle. The overall annual incidence of childhood cardiomyopathies is estimated at about 1 per 100,000 children and is significantly higher during the first 2 years of life. Dilated cardiomyopathies account for approximately half of the cases. Hypertrophic cardiomyopathies form the second largest group, followed by the less common left ventricular non-compaction and restrictive phenotypes. Infectious, metabolic, genetic, and syndromic conditions account for the majority of cases. Congestive heart failure is the typical manifestation in children with dilated cardiomyopathy, whereas presenting symptoms are more variable in other phenotypes. The natural history is largely influenced by the type of cardiomyopathy and its underlying aetiology. Results from a national population-based study revealed 10-year transplant-free survival rates of 80, 62, and 48% for hypertrophic, dilated and left ventricular non-compaction cardiomyopathies, respectively. Long-term survival rates of children with a restrictive phenotype have largely been obscured by early listing for heart transplantation. In general, the majority of adverse events, including death and heart transplantation, occur during the first 2 years after the initial presentation. This review provides an overview of childhood cardiomyopathies with a focus on epidemiology, natural history, and outcomes.

## Introduction

**Figure 1:**
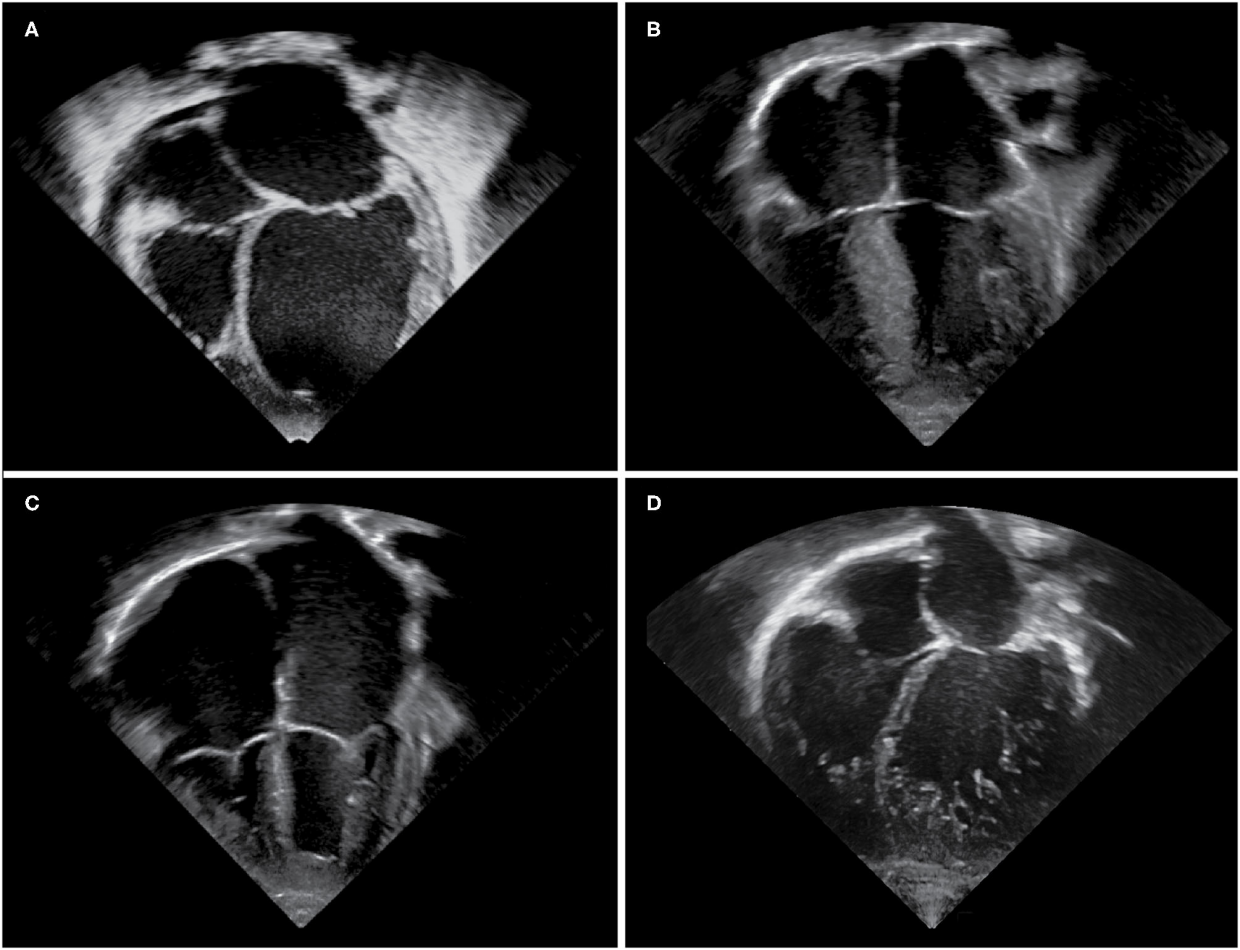
Echocardiographic images of cardiomyopathy phenotypes. Apical four chamber views demonstrating **(A)** a dilated left ventricle and left atrium in a DCM patient, **(B)** hypertrophy of the interventricular septum and left ventricular free wall in a HCM patient, **(C)** massively dilated atria and small right and left ventricular cavities in an RCM patient, **(D)** an extensively trabeculated myocardium with a compacted and non-compacted layer and deep intertrabecular recesses most prominent at the left ventricular apex and free wall in an LVNC patient.

Paediatric cardiomyopathies form an uncommon and heterogenous group of disorders, which are characterised by structural, mechanical, and electrical abnormalities of the heart muscle (1, 2). Aetiologies are diverse, and include infections, toxin exposure, tachyarrhythmias, genetic mutations, and underlying metabolic or neuromuscular disorders (1–6). Large population registries together with national and multicentre studies have contributed considerably to the increasing knowledge on epidemiology and outcomes of childhood cardiomyopathies (5, 7–24). The overall annual incidence is estimated at about 1 per 100,000 children, with a significantly higher incidence during the first 2 years of life (7, 17, 18). Dilated and hypertrophic cardiomyopathies are the most common types, whereas left ventricular non-compaction and restrictive cardiomyopathy occur less frequently (7, 17). Arrhythmogenic ventricular cardiomyopathy is rarely diagnosed during childhood and will not be discussed in this article. The terms dilated, hypertrophic, restrictive, and non-compaction depict different phenotypes (see Figure 1) and thereby assist in grouping cardiomyopathies, however they do not describe specific disease entities. The European Society of Cardiology classifies cardiomyopathies according to their predominant phenotype, but does not recognise left ventricular non-compaction as a separate entity, whereas the American Heart Association classifies cardiomyopathies according to their aetiology, with left ventricular non-compaction considered to be a separate entity (25, 26). Some children may present with a mixed phenotype, and cardiomyopathy phenotypes may undulate or transition during the course of disease (1, 12, 13, 27). This review summarises the most common forms of paediatric cardiomyopathies, with a focus on epidemiology and natural history.

## Dilated Cardiomyopathy

### Aetiologies

Dilated cardiomyopathy (DCM) is characterised by left ventricular dilation and systolic dysfunction. Important aetiologies in childhood include infections, toxic causes (including chemotherapy), genetic mutations, and other causes such as inborn errors of metabolism and neuromuscular disorders (1). Recent advances in genetic diagnostics, including the introduction of next-generation DNA sequencing technologies and extended cardiomyopathy gene panels, have increased the detection rate for pathogenic mutations in adult DCM patients to about 40% (28, 29). Sarcomere gene mutations are thought to be responsible for 35–40% of genetic cases (1). Evidence of myocarditis has been found in up to one third of children with DCM undergoing early endomyocardial biopsy (20, 21, 30). Individuals with variants in genes coding for cardiac structural proteins may be particularly susceptible to severe myocarditis (31, 32). However, despite significant diagnostic progress over the last decade, the aetiology of childhood DCM often remains unknown.

### Epidemiology

DCM is the most common type of childhood cardiomyopathy. It comprised about half of all cases in the Paediatric Cardiomyopathy Registry (PCMR), a large multicentre North American study, as well as in the National Australian Childhood Cardiomyopathy Study (NACCS), a population-based cohort study which included children younger than 10 years of age at diagnosis. The overall incidence was 0.58–0.73 per 100,000 children and varied significantly by age (see Table 1). In both studies, the highest annual incidence was observed during the first year of life (see Table 1) (7, 17). A nationwide cardiomyopathy study from Finland, which included only idiopathic cardiomyopathy cases, demonstrated an overall DCM incidence of 0.34 per 100,000 children up to 20 years and an 11-fold higher incidence during the first year of life (see Table 1) (18). A large prospective study undertaken in the United Kingdom and Ireland assessed the incidence of paediatric new-onset heart failure due to heart muscle disease of known and unknown aetiologies. The authors reported an incidence of 0.76 symptomatic DCM cases per 100,000 children under 16 years (19). Lymphocytic myocarditis accounted for 22% of all cases in this study, and comprised 16–36% of DCM cases in the PCMR and NACCS (19–21). Endomyocardial biopsy is not universally performed in newly diagnosed cases of DCM and the presence of positive histological findings for lymphocytic myocarditis decreases rapidly within weeks after presentation, which may lead to an underestimation of inflammatory DCM in children (21, 33).

**Table 1:** Annual incidence and median age at diagnosis for each type of cardiomyopathy (CM).

**Type of CM**	**Age range at diagnosis**	**Number of cases**		**Median age at diagnosis**	**Annual incidence per 100,000 children**		**References**
		**All cases**	**Age at diagnosis <1 y**		**All cases**	**Age at diagnosis <1 y**	
DCM	0–18 y	239	–	1.8 y	0.58	4.58	PCMR (7)
	0–10 y	184	121	7.5 m	0.73	4.76	NACCS (17)
	0–20 y	56	29	1.1 y	0.34	3.8	Finland study (18)
HCM	0–18 y	196	–	5.9 y	0.47	3.2	PCMR (7)
	0–10 y	80	48	5.7 m	0.32	1.89	NACCS (17)
	0–20 y	40	2	13 y	0.24	0.26	Finland study (18)
RCM	0–10 y	8	0	3 y	0.03	0	NACCS (17)
	0–20 y	6	0	7.2 y	–	0	Finland study (18)
Other^a^	0–18 y	15	–	–	0.04	–	PCMR (7)
Unclassified^b^	0–10 y	42	30	3.8 m	0.17	1.18	NACCS (17)
Total	0–18 y	467	193	–	1.13	8.34	PCMR (7)
	0–10 y	314	199	–	1.24	7.84	NACCS (17)
	0–20 y	107	31	–	0.65	4.1	Finland study (18)

a *Includes restrictive and other identified types of CM*.

b *Includes LVNC (29 of 42 cases)*.

### Presenting Features

The majority of children with DCM, particularly infants, present with varying severity of congestive cardiac failure, with symptoms ranging from feeding difficulties to cardiovascular collapse, and rarely sudden death. Older children with a positive family history and those with neuromuscular disorders or inborn errors of metabolism may be diagnosed as part of routine screening, prior to symptom onset. Lymphocytic myocarditis has a phenotype that may be indistinguishable from idiopathic DCM. A history of a prior viral illness is not always present and may be misleading (34). Data from the PCMR and NACCS showed that at the time of diagnosis, 71–90% of children with DCM had clinical evidence of congestive heart failure (20, 21). In the NACCS DCM cohort, 5% presented with sudden cardiac death, 2% with exercise intolerance or arrhythmia, and 3% for routine family screening. Intensive care unit admission was required in 45% and inotropic support was administered in 40% (21). Significant comorbidities associated with cardiomyopathy related intensive care unit admissions included renal failure, thromboembolic events and hepatic impairment (35).

### Natural History

Outcomes for children with DCM are highly variable, ranging from complete recovery to death or requirement for transplantation. Beyond infancy, DCM is the most common indication for heart transplantation in children (36). Data from the PCMR and NACCS demonstrated an overall 5 year transplant-free survival of 54–65 % (see Table 2) (20, 21). Risk stratification helps to identify individuals who can be treated medically and those who require advanced heart failure therapy including listing for heart transplantation. Transplant-free survival analysis according to the underling aetiology from the PCMR is shown in Table 2 (20). Long-term outcome data from the NACCS revealed transplant-free survival of 56% after 20 years (22). The highest risk was observed early after diagnosis, with a 26% risk of death or transplantation within the first year and only 1% per year thereafter (22). Similar results were found in the UK study of children admitted with new-onset heart failure secondary to dilated cardiomyopathy. Event-free survival at 1 year was only 66 and 16% had undergone heart transplantation within the first year following diagnosis (19).

**Table 2:** Transplant-free survival for each type of cardiomyopathy (CM).

**Patient characteristics**				**Transplant-free survival rate after diagnosis**			**References**
**Type of CM**	**Subgroups**	**Age range at diagnosis *(median age)***	**Number of cases**	**1 y**	**5 y**	**10 y** ***(>10 y)***	
DCM	All	0–10 y	175	74%	65%	62% *(20 y: 56%)*	NACCS (22)
	All	0–18 y *(1.54 y)*	1,426	69%	54%	46%	PCMR (20)
	Idiopathic		941	61%	47%	42%	
	Myocarditis		222	79%	73%	60%	
	Familial		66	81%	59%	59%	
	IEM		54	84%	78%	78%	
	MFS		15	91%	76%	76%	
	NMD		125	83%	52%	26%	
HCM	All	0–10 y *(0.48 y)*	80	86%	–	80% (*20 y: 78%)*	NACCS (8)
	All	0–18 y	855	–	–	–	PCMR (23)^a^
	Idiopathic	*(7.07 y)*	634	94.4 %	89.8%	85.3%	
	IEM	*(0.42 y)*	74	53.6%	41.7%	–	
	MFS	*(0.41 y)*	77	82.4%	74.4%	74.4%	
	NMD	*(10.10 y)*	64	98.2%	98.2%	91.7%	
	All	0–16 y *(5.2 y)*	687	95.6%	90.6%	86.3%	UK study, Norrish et al. (6)
	Non-syndromic		433	97.6%	92.7%	87.5%	
	RASopathy		126	92.5%	90.5%	85.9%	
	IEM		64	82.2%	66.4%	66.4%	
	FRDA		59	100%	97.1%	97.1%	
RCM	All	0–18 y	152	–	–	–	PCMR (10)
	Pure RCM	*(6.1 y)*	101	48%	22%	–	
	RCM/HCM	*(6.3 y)*	51	65%	43%	–	
LVNC	All	0–10 y *(0.3 y)*	29	69%	52%	48% *(15 y: 45%)*	NACCS (13)
	All	0–14 y *(9.3 y)*	242	–	–	–	USA study, Brescia et al. (57)
	Dilated		46	–	63%	–	
	Hypertrophic		66	–	86%	–	
	Mixed		68	–	64%	–	
	Pure		62	–	98%	–	

*IEM, inborn errors of metabolism; MFS, malformation syndromes; NMD,neuromuscular disorders; FRDA, Friedreich's ataxia*.

a *Numbers represent overall survival rates by aetiologies, transplantation status not further specified*.

Other risk factors in children with DCM relate to age at diagnosis and the severity of cardiac dysfunction. Risk factors for death or transplantation in the NACCS comprised age below 4 weeks or above 5 years at presentation, familial dilated cardiomyopathy, a lower initial fractional shortening *z*-score and failure to improve fractional shortening *z*-score during follow-up (21, 22). Favourable outcomes including transplant-free survival and reverse remodelling were more frequent in children with lymphocytic myocarditis compared to other aetiologies, with echocardiographic normalisation of LV function found in 92% during follow-up (22). Although less frequent, reverse modelling has also been observed in children without proven or suspected myocarditis. Everitt et al. reported echocardiographic recovery of LV function within 2 years in 22% of cases with idiopathic DCM included in the PCMR. Younger age (<10 years) and less LV dilation at diagnosis were independent predictive factors of echocardiographic normalisation. Some patients developed recurrence of congestive cardiac failure following initial echocardiographic normalisation (37). Persistence of increased levels of N-terminal proBNP after initial stabilisation was found to be predictive for the risk of cardiac death in children with idiopathic DCM (38).

Pahl et al. reviewed risk factors for sudden cardiac death (SCD) in DCM from the PCMR and found a 5-year incidence of 3%. Younger age at diagnosis (<14.3 y), systolic LV dilation (LV end systolic dimension *z*-score >2.6) and posterior wall thinning were identified as risk factors for sudden death (39).

The overall survival for childhood DCM in North America has improved in the most recent era, despite rates of echocardiographic normalisation and cardiac transplantation that did not differ by comparison with a previous era (40). This may be due to improved resuscitation and/or the use of adult-based chronic heart failure therapies.

## Hypertrophic Cardiomyopathy

### Aetiologies

Hypertrophic Cardiomyopathy (HCM) is a condition characterised by left or biventricular hypertrophy in the absence of structural heart disease or increased ventricular afterload. Sarcomeric mutations represent the most common genetic cause in children and adults with a detection rate of about 60% in childhood HCM diagnosed beyond the first year of life, similar to that of adult HCM patients (41). By comparison with adult HCM, childhood HCM comprises a much more heterogenous group with diverse aetiologies and spectrum of disease (6). Genetic causes include inborn errors of metabolism, malformation syndromes (primarily RASopathies), neuromuscular disease as well as pathogenic mutations in genes coding for sarcomeric proteins. Typical causes within these four categories are Pompe disease, Noonan syndrome, Friedreich ataxia, and MYBPC3 or MYH7 mutations, respectively (1, 23).

### Epidemiology

HCM forms the second commonest group of childhood cardiomyopathies, comprising 25–42% of all cases, respectively. The overall annual incidence is 0.24–0.47 per 100,000 children (see Table 1) (7, 17, 18). HCM caused by inborn errors of metabolism and malformation syndromes generally presents in infancy and contributes significantly to an early first peak in incidence. A second, smaller peak during adolescence and early adult life is largely due to sarcomeric protein mutations (1, 41). Data from the NACCS, which included children only aged 0–10 year at diagnosis and excluded those with metabolic and neuromuscular conditions, demonstrated a median age of 5.7 months at presentation. The NACCS and PCMR reported a marked decline in incidence between the first year and subsequent years of life (see Table 1). In the Finnish study, which also excluded patients with metabolic and neuromuscular aetiologies but included children up until 20 years at diagnosis, the median age at diagnosis was 13 years, and 39% of patients were over 15 years of age at presentation (see Table 1).

### Presenting Features

The clinical status at presentation ranges from asymptomatic to symptoms of exercise intolerance, chest pain, palpitations, syncope, or cardiac arrest (1). Congestive heart failure or arrhythmic symptoms are found in 10–15% of cases at presentation (6, 24). Children with inborn errors of metabolism and malformation syndromes generally present earlier, and are more likely to have congestive heart failure at the time of diagnosis (23). Aborted sudden cardiac death or out of hospital arrest are an uncommon initial presentation in childhood HCM (6).

### Natural History

The natural history and outcome of childhood HCM largely depend on the age at presentation and the underlying aetiology. The highest risk of mortality is seen in those diagnosed during the first year of life (24).

Overall survival free from death or transplantation was found to be about 90% at 5 years and 78% at 20 years from presentation (see Table 2) (6, 8, 24). The risk of mortality or transplantation was 14% during the first year after presentation, decreasing to 0.4% per subsequent year (8).

The worst outcomes are in infants with heart failure at the time of diagnosis and in older children with marked restrictive pathophysiology. Other risk factors include concentric left ventricular hypertrophy at diagnosis, Noonan syndrome, and increasing LV ventricular free wall thickness and worsening LV systolic function during follow-up (8). In the PCMR, children with non-syndromic HCM diagnosed before 1 year of age had a higher mortality, which reduced in those surviving infancy (23). Similarly, Noonan syndrome patients had a markedly reduced 1-year survival when diagnosed with congestive heart failure before 6 months of age (42). The highest 5 year survival rate was observed in HCM secondary to neuromuscular disease (see Table 2) (23).

While congestive heart failure accounts for the majority of early deaths in childhood HCM, the most common mode of death overall is SCD (6, 8). Arrhythmic events have been observed with a rate of 1.2 per 100 patient years in a large UK study, with more frequent occurrence in non-syndromic patients (6). The identification of specific paediatric risk factors for SCD is essential to guide implantable cardioverter defibrillator (ICD) insertion in a population that frequently experiences no cardiac symptoms in their daily life. A systematic review and meta-analysis of clinical risk factors for sudden cardiac death in childhood cardiomyopathy identified previous adverse cardiac events, non-sustained ventricular tachycardia, syncope, and extreme left ventricular hypertrophy as major factors (43). Norrish et al. recently described a novel risk prediction model for SCD in childhood HCM, with the objective of providing individualised risk estimates. Unexplained syncope, maximal left ventricular wall thickness, left atrial diameter, and non-sustained VT were found to have the strongest association with the composite outcome of SCD or an equivalent event (44). Miron et al. also described an SCD risk prediction model for paediatric HCM. This group used the above mentioned four risk factors as well as age at diagnosis for a clinical model and added the presence of a pathogenic gene variant for a combined clinical/genetic model (45). The relationship between left ventricular outflow obstruction and sudden death is complex, with some studies showing either a protective effect or an inverse relationship in children with the highest gradients (44–46). Arrhythmic events leading to sudden cardiac death continue to occur in adult cohorts at about 0.7% per year (47).

## Restrictive Cardiomyopathy

### Aetiologies

Restrictive cardiomyopathy (RCM) is a rare form of heart muscle disease defined by “normal or decreased volume of both ventricles associated with biatrial enlargement, normal left ventricular wall thickness and atrioventricular valves, impaired ventricular filling with restrictive physiology, and normal (or near normal) systolic function” (26). Nearly a quarter of patients with RCM have a family history of cardiomyopathy (10). An increasing number of genetic mutations in sarcomeric and non-sarcomeric proteins have been reported, providing evidence of overlap with other forms of cardiomyopathy (5, 48, 49). A significant subgroup of RCM cases has a mixed phenotype, most commonly combining characteristics of RCM and HCM (10). RCM has also been described in association with inborn errors of metabolism, infiltrative disease, and skeletal myopathy (1, 50).

### Epidemiology

RCM is the rarest form of paediatric cardiomyopathy with an incidence of 0.03–0.04 per 100,000 children in Australia and the United States (see Table 1) (7, 17). RCM accounted for 2.5% of cases in the NACCS, and the PCMR reported 3% of pure RCM cases and an additional 1.5% of mixed RCM/HCM phenotype cases (7, 17). Age at diagnosis ranges from early infancy to late adulthood (1). Unlike other childhood cardiomyopathies, RCM becomes more frequent with increasing age. Only 10% of pure RCM cases in the PCMR were diagnosed during the first year of life (10).

### Presenting Features

Early symptoms of RCM may be non-specific, including general fatigue and exercise intolerance. Clinical findings secondary to elevated systemic and pulmonary venous pressures include peripheral oedema, hepatomegaly, pulmonary oedema, and pulmonary hypertension (1). In the later stages of disease, patients may develop systolic dysfunction (1). Syncope is a non-specific but ominous presenting symptom, which may be caused by arrhythmias, coronary ischemia, or thromboembolic events (51).

### Natural History

Whilst RCM has the worst outcomes of any childhood cardiomyopathy, the natural history has largely been obscured by early referral for transplantation. Long-term transplant-free survival data is therefore sparse. Because of its relentless and progressive nature, patients are at risk of sudden death, congestive heart failure, atrial and ventricular arrhythmias, conduction disorders and thromboembolism (1, 52). Transplantation free survival for children with pure RCM in the PCMR was 48 and 22% 1 and 5 years after diagnosis, respectively (10). The mixed phenotype group (RCM/HCM) demonstrated a 2-fold higher 5 year transplant-free survival (see Table 2). Overall freedom from death after 5 years was identical for both cohorts, indicating a preference for earlier transplantation in pure RCM patients (10). Russo et al. reviewed 21 cases of RCM in a single centre retrospective analysis, and reported transplantation free survival of 80.5 and 20% at 1 and 10 years, respectively (53). Anderson et al. analysed their institutional experience for children with RCM comparing a historical cohort of 9 cases, diagnosed between 1975 and 1993, with a contemporary cohort of 12 cases. Transplantation free survival over 5 years was 38% in both groups, however overall survival in the contemporary group was 80 vs. 38% in the historical group (54).

Due to the infrequence of childhood RCM and small study cohorts, assessment of risk factors for outcome has proven difficult, and results have been inconsistent. In the PCMR, heart failure symptoms and lower fractional shortening *z*-score at diagnosis were identified as independent risk factors for decreased transplant-free survival (10). Similarly, higher initial echocardiographic left atrial dimensions and a requirement for diuretics during follow-up have been associated with increased mortality (49). Anderson et al. observed an association of marked elevation of mitral valve Doppler E/e′ ratio on echocardiography with increased mortality (54). Rivenes et al. evaluated risks factors predictive of sudden death and cardiovascular collapse in 18 children with RCM from a single centre retrospective study. They reported an increased risk of ischemia-related complications and mortality in the entire patient group. The risk of sudden death was highest in girls with clinical signs suggestive of ischemia, in particular chest pain and syncope at presentation. The subgroup at risk of sudden death appeared well and had no clinical evidence of ongoing congestive heart failure (51). Walsh et al. observed that PR prolongation and a wider QRS complex on a baseline ECG were associated with an increased incidence of acute cardiac events, and they found a substantial risk for acute high-grade heart block in RCM patients (52). An elevated pulmonary vascular resistance is present in up to 40% of children with RCM and may impact on the timing for transplant referral (11). Serial cardiac catheterisation is often undertaken to detect this serious complication which may impact on transplant suitability (5, 11).

## Left Ventricular Non-Compaction

### Aetiologies

Left ventricular non-compaction (LVNC) is a heterogenous form of cardiomyopathy characterised by excessive trabeculation of the left or both ventricles with deep intertrabecular recesses, most frequently affecting the left ventricular apex. LVNC was classified as a separate cardiomyopathy by the American Heart Association in 2006 (26) however there is ongoing discussion about whether it is a distinct entity or a morphological phenotype (55). Arrest in normal endomyocardial morphogenesis with failure of trabecular compaction is thought to be causative especially in paediatric cases (3). A similar phenotype can manifest at any time in adult life secondary to conditions associated with an increased left ventricular preload (56). LVNC can be isolated or associated with other cardiomyopathy phenotypes, arrhythmias, or congenital heart disease. LVNC is commonly found in Barth syndrome, an X-linked recessive disorder caused by tafazzin gene mutations, and has also been reported in patients with inborn errors of metabolism, neuromuscular diseases, and genetic syndromes (1). Genetic testing detects variants in 30–45% of cases, with sarcomeric mutations found most frequently (3, 56).

### Epidemiology

The rates of diagnosis of LVNC in children have increased during the last decades, which is thought to reflect increased awareness and improved imaging techniques rather than a rise in incidence (12). Data from the NACCS demonstrated an incidence of 0.11 per 100,000 children aged 0–10 years, and a 7-fold higher incidence in infants (see Table 1). LVNC was found in 9.2% of children diagnosed with cardiomyopathy under 10 years (17). In the PCMR, LVNC was present in 4.8% of cases. LVNC was found to be associated with dilated, hypertrophic and indeterminate phenotypes in 59, 11, and 8% of cases, respectively, and isolated LVNC occurred in the remaining 23% of cases. The median age at diagnosis was significantly higher in isolated LVNC (9.8 years) compared to cases with mixed phenotypes (0.4–0.6 years) (12).

### Presenting Features

Patients with LVNC may be found on routine screening but may also present with thromboembolic events, arrhythmias, or congestive heart failure (3). The variability of presenting symptoms reflects the phenotypic diversity, and associated features of other types of cardiomyopathies contribute significantly to the clinical picture. In the largest cohort of paediatric LVNC patients, of which almost 40% were infants, 25% presented primarily with congestive heart failure, 17% with arrhythmias, 19% with a heart murmur, and 37% were asymptomatic (57). A positive family history of cardiomyopathy was present in 23% of all cases, however, only 25% of this subgroup had a family history of LVNC.

### Natural History

The outcome of paediatric LVNC is highly variable and depends on the underlying pathophysiology (1). Brescia et al. reviewed the risk of mortality and sudden death in the largest published cohort of paediatric LVNC patients. They found a strong relationship between the identified phenotype and the risk of death or transplantation. Five-year transplant-free survival rate was excellent for the normal-dimension phenotype, intermediate for the hypertrophic phenotype and worst for the dilated and mixed phenotypes (see Table 2). The greatest risk factors for death or transplantation were the presence of systolic dysfunction and/ or arrhythmias. Sudden cardiac death occurred in 6.2% of cases over a 19-year study period, with systolic dysfunction present in 95% and documented arrythmia in 60% of these patients. Early presentation during the first year of life was an additional independent risk factor, with a 25% risk of death or transplantation in infantile LVNC (57). Jefferies et al. similarly observed the worst outcome for patients with LVNC and the dilated or indeterminate phenotype in the PCMR. The risk of death was highest in the first year after diagnosis (12). Shi et al. reviewed long-term outcomes of children with LVNC from the NACCS. This cohort included mainly young and severely affected infants, with a median age of 0.3 years at diagnosis and congestive heart failure present in 83% at the time of diagnosis. Freedom from death and transplantation was 45% at 15 years after diagnosis (see Table 1). Propensity score matching suggested a 2-fold higher risk of death and transplantation for patients with LVNC and a dilated phenotype compared to children with DCM from the same registry (13). Children with an isolated LVNC phenotype and without observable cardiac dysfunction have been found to have a favourable outcome (12). However, progression to an associated cardiomyopathy phenotype with a risk of mortality has been observed in a small proportion of cases, therefore ongoing surveillance is recommended (12).

## Discussion

Over the last 2 decades, registries and national studies have provided important data on epidemiology and outcomes of childhood cardiomyopathies. The reported overall annual incidence was between 0.65 and 1.24 per 100,000 children (7, 17, 18). Consistently throughout these epidemiological studies, the annual incidence of cardiomyopathy during the first year of life was 6–7 times higher than the above-mentioned average incidences (7, 17, 18). This peak incidence during infancy was found in all types of cardiomyopathy except for RCM which typically presents later in childhood (7, 17, 18). PCMR data demonstrated a small second peak during adolescence which was related to HCM and cardiomyopathies secondary to neuromuscular diseases (7).

LVNC cardiomyopathy has increasingly gained attention over the last two decades and there is ongoing discussion with regards to its diagnostic criteria (25, 26). Increasing rates of diagnosis reflect an increasing awareness of this entity compared to prior eras (12).

While there has been a dramatic improvement in survival of congenital heart disease over the last decades, the overall outcomes for childhood cardiomyopathies remain unfavourable (58, 59). Recovery from inflammatory cardiomyopathies or successful rhythm control in arrhythmogenic heart failure represent exemptions. Childhood cardiomyopathies comprise the most common indication for cardiac transplantation beyond the first year of age (36). The highest risk of death or cardiac transplantation occurs during the first year after diagnosis (19, 22). Knowledge of the natural history and risk factors for adverse outcomes, gained from registries and large multicentre studies, assists in risk stratification and case selection for advanced heart failure therapies, and for ICD insertion for prevention of sudden cardiac death.

## Conclusion

Paediatric cardiomyopathies, although rare, carry a substantial burden of disease due to the risk of morbidity and mortality and a lack of curative therapy. Established registries have provided valuable insights into the natural history and risk factors, assisting in decision-making on sudden cardiac death prevention and cardiac transplantation.

## Author Contributions

AR drafted the manuscript and designed figures and tables. RW supervised the project and contributed substantially to the final version of the manuscript. Both authors contributed to the article and approved the submitted version.

## Conflict of Interest

The authors declare that the research was conducted in the absence of any commercial or financial relationships that could be construed as a potential conflict of interest.

## Publisher's Note

All claims expressed in this article are solely those of the authors and do not necessarily represent those of their affiliated organizations, or those of the publisher, the editors and the reviewers. Any product that may be evaluated in this article, or claim that may be made by its manufacturer, is not guaranteed or endorsed by the publisher.
